# Desert soil clay content estimation using reflectance spectroscopy preprocessed by fractional derivative

**DOI:** 10.1371/journal.pone.0184836

**Published:** 2017-09-21

**Authors:** Jingzhe Wang, Tashpolat Tiyip, Jianli Ding, Dong Zhang, Wei Liu, Fei Wang, Nigara Tashpolat

**Affiliations:** 1 College of Resources and Environment Science, Xinjiang University, Urumqi, Xinjiang, China; 2 Key Laboratory of Oasis Ecology, Xinjiang University, Urumqi, Xinjiang, China; ICAR-Central Arid Zone Research Institute, INDIA

## Abstract

Effective pretreatment of spectral reflectance is vital to model accuracy in soil parameter estimation. However, the classic integer derivative has some disadvantages, including spectral information loss and the introduction of high-frequency noise. In this paper, the fractional order derivative algorithm was applied to the pretreatment and partial least squares regression (PLSR) was used to assess the clay content of desert soils. Overall, 103 soil samples were collected from the Ebinur Lake basin in the Xinjiang Uighur Autonomous Region of China, and used as data sets for calibration and validation. Following laboratory measurements of spectral reflectance and clay content, the raw spectral reflectance and absorbance data were treated using the fractional derivative order from the 0.0 to the 2.0 order (order interval: 0.2). The ratio of performance to deviation (RPD), determinant coefficients of calibration ($R_{c}^{2}$), root mean square errors of calibration (RMSEC), determinant coefficients of prediction ($R_{p}^{2}$), and root mean square errors of prediction (RMSEP) were applied to assess the performance of predicting models. The results showed that models built on the fractional derivative order performed better than when using the classic integer derivative. Comparison of the predictive effects of 22 models for estimating clay content, calibrated by PLSR, showed that those models based on the fractional derivative 1.8 order of spectral reflectance ($R_{c}^{2}$ = 0.907, RMSEC = 0.425%, $R_{p}^{2}$ = 0.916, RMSEP = 0.364%, and RPD = 2.484 ≥ 2.000) and absorbance ($R_{c}^{2}$ = 0.888, RMSEC = 0.446%, $R_{p}^{2}$ = 0.918, RMSEP = 0.383% and RPD = 2.511 ≥ 2.000) were most effective. Furthermore, they performed well in quantitative estimations of the clay content of soils in the study area.

## Introduction

Direct measurements of various physical and chemical properties of soil are more accurate than estimations via remote sensing methods; however, they often require intensive field investigations that can be restricted by limited funds and labor [1]. Remote sensing is considered a promising alternative approach to conventional methods for estimating soil properties because of its high efficiency, low cost, and its large-scale, non-destructive, rapid data acquisition [1, 2]. In particular, its characteristics of high spectral resolution, convenience and controlled condition are well suited to laboratory analysis of soil spectral reflectance. Traditionally, the measurement of clay content in soil is complicated and it requires more chemical reagents and caution, especially for salt-affected soils [3]. Therefore, based on the different spectral responses in the VIS–NIR (visible and near-infrared) bands to soil particle size, spectral analysis technology could be used as an alternative to ensure the accurate estimation of the clay content in soil.

Many studies have been conducted on the spectral response features and quantitative prediction of clay content [4–7]. For example, Ben-Dor and Banin [8] considered that clay content was correlated strongly with the clay minerals in soil, and that the principal characteristic bands were related to the lattice hydroxyl groups of layered silicates. Stenberg et al. [9] reviewed the application of VIS–NIR spectroscopy in soil science, and their results showed that the characteristic bands cover the absorption spectra of the clay content (1400 nm), hydroxyl groups (1900 nm) and clay minerals (2200 nm). Using VIS–NIR spectroscopy and pretreatment by Savitzky–Golay (SG) smoothing, first derivative with SG smoothing, and other mathematical methods, the prediction performances of models based on multivariate adaptive regression splines were improved [10]. In order to obtain better accuracy in estimations of clay and soil organic matter (SOM) contents, Nawar et al. [11] applied the first- and second-derivative and another seven algorithms to pretreat the reflectance data.

The pretreatment of spectral reflectance is efficient in terms of improving the accuracy of spectral estimation models. In previous research, spectral reflectance has been transformed often by some commonly used functions, e.g., absorbance and the corresponding integer derivative algorithms [10, 12, 13]. To some degree, the spectral derivative can eliminate the background influence of the environment and highlight certain spectral features [14]. However, because the quantity of information is considerable, the pretreatment of spectral reflectance by a general integer order derivative might influence the detection of crucial information and, to some extent, cause loss of spectral information [15]. Fractional calculus is a theoretical branch of mathematics that generalizes the classic integer derivative into an arbitrary (non-integer) order, which has broadened the concept of the classic integer derivative [14, 16]. Because of its improved accuracy and higher efficiency, it has been used widely in system control and diagnosis, digital filtering, signal and image processing, and other related fields [15, 17–19]. Of particular relevance, the fractional derivative has been applied to the pretreatment of the spectral data of saline soil [20], which has demonstrated its validity in detecting spectral information from reflectance data of soil from arid regions.

Compared with free iron, clay content is a more reliable indicator of the age and weathering degree of soil at the various stages of development [21]. Soil salinization and desertification are the most common but serious environmental problems in the Ebinur Lake basin of Northwest China [22]. Therefore, the calibration of a rapid and accurate model for the quantitative estimation of local soil clay content is crucial. Given this context and motivated by previous research, the objective of this study was to use laboratory-derived spectral reflectance data pretreated by the fractional derivative, in combination with known soil clay content to establish an acceptably accurate and stable model for soil parameter estimation.

## Materials and methods

### Study site and sampling

Overall, 103 soil samples were collected from the study area, namely, the Ebinur Lake basin in the southwest of the Junggar Basin in the Xinjiang Uighur Autonomous Region of China (44°30′–45°16′N, 82°06′–83°40′E). The study area has an arid desert climate with mean annual precipitation, potential evapotranspiration, and temperature of 102 mm, 2492 mm, and 7.2°C, respectively [23, 24]. The Alataw Pass is a famous entrance for the northwest wind in the Ebinur Lake region. On average, winds with speeds >8 m s^-1^ occur on 164 days per year, reaching a maximum of 185 days per year [25, 26]. The main geomorphic types are stone desert, gravel desert, salt desert, and swamp. The soil types are mainly Mollic Solonchaks, Gypsic Regosols, and Stagnic Solonetz [22, 27]. Soil erosion by wind is a common phenomenon within this region because of the extreme weather and particular texture of the soil.

**Fig 1:**
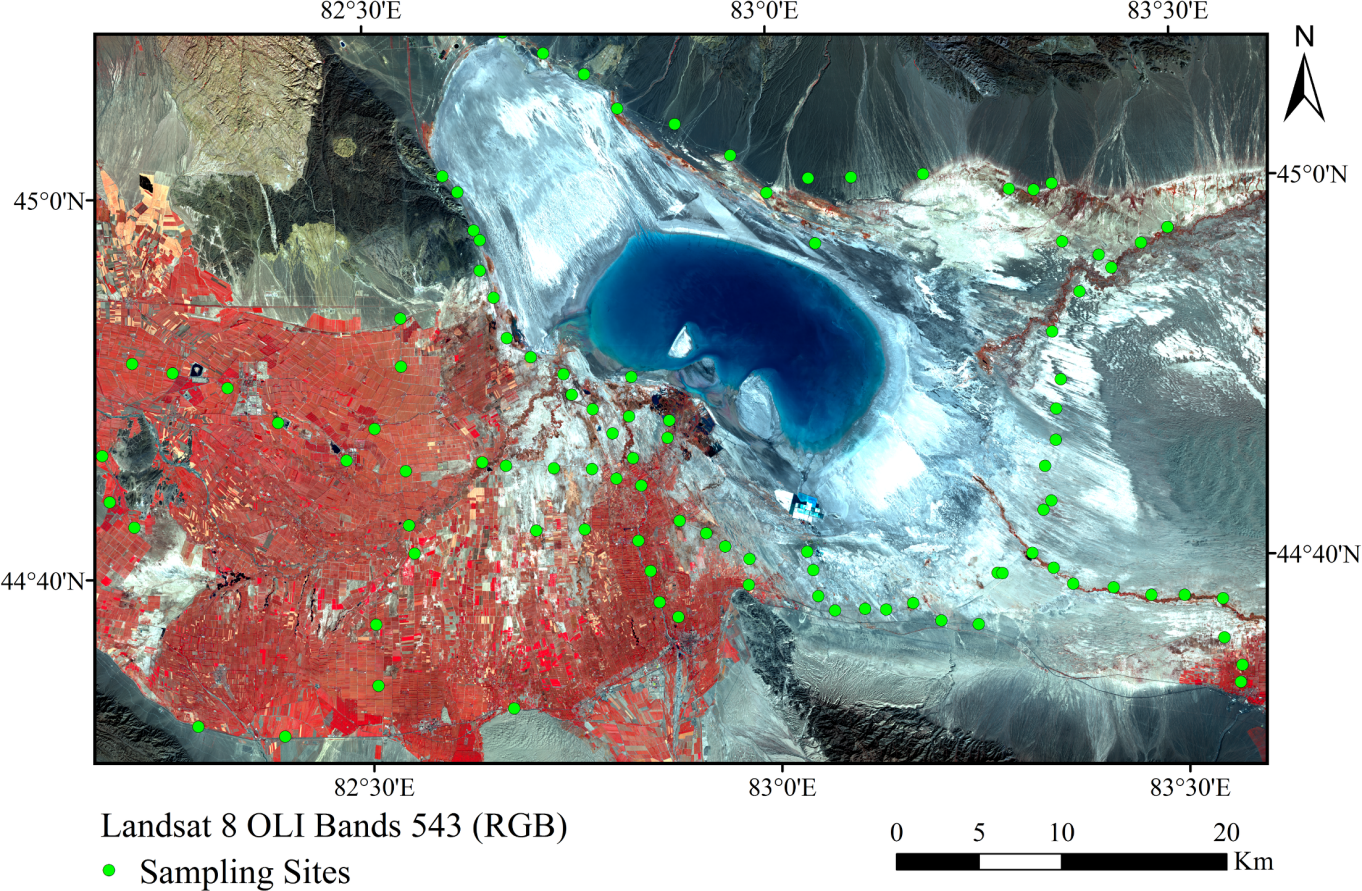
Study area with soil sample locations.

In order to obtain representative soil samples, 103 sampling sites (30 × 30 m) were established, with consideration of the typical landforms, landscape types, and soil textures of the study area. Within each site, soil samples were collected at five evenly distributed points and then mixed thoroughly to obtain a representative sample. Overall, 103 soil samples were collected at depths of 0–10 cm from the study area during May 18–29, 2015 (Fig 1).

### Laboratory analysis

All 103 soil samples were air-dried, crushed, and then passed through a 2.0 mm sieve and the resulting fine earth (<2.0 mm) was retained for further analysis. The potassium dichromate method was applied for the measurement of SOM content [28]. The concentrations of K^+^ and Na^+^ were determined using the flame photometry method, and those of Ca^2+^ and Mg^2+^ were determined using the EDTA complexometric titration method [28]. The soil electrical conductivity (EC) was determined using a WTW inoLab® Multi 3420 Set B multiparameter measuring instrument (Wissenschaftlich-Technische Werkstätten GmbH, Germany) with extracts of soil and distilled water in a ratio of 1:5. Soil clay content was determined using a particle analyzer and imaging system (Bluewave S3500, Microtrac Inc., Largo, FL, USA) at room temperature.

### VIS–NIR spectroscopy and spectral processing

#### Spectral measurement

For controlled irradiance conditions, the measurements of spectral reflectance for all soil samples were conducted in a dark laboratory. The reflectance spectra were measured using an ASD FieldSpec® 3 portable spectrometer (Analytical Spectral Devices Inc., Boulder, CO, USA) with a spectral range of 350–2500 nm. The sampling intervals of this spectrometer are 1.4 nm (350–1000 nm) and 2.0 nm (1000–2500 nm), while the resampling interval is 1.0 nm [5, 20]. Circular containers with a diameter of 12.0 cm and a depth of 1.8 cm were used to store the soil samples (1.5 cm is considered optically infinitely thick for soil). To avoid contamination during the measurements, these containers had been painted black in advance [29]. Notably, each sample had the same flat measuring surface [30]. Scanning was performed using a fiber optic sensor with an 8° zenith angle, which was placed 10.0 cm above the samples [20]. For lighting, a halogen lamp (50 W) was placed 50.0 cm from each sample at a zenith angle of 30° [1, 11]. For each measurement, 20 spectral curves were gathered from the central area of the sample, and the final reflectance was yielded by averaging these 20 representative spectra. To ensure accuracy, the spectrometer was calibrated using a Spectralon® panel with 100% reflectance prior to each measurement.

#### Spectral preprocessing

**Fig 2:**
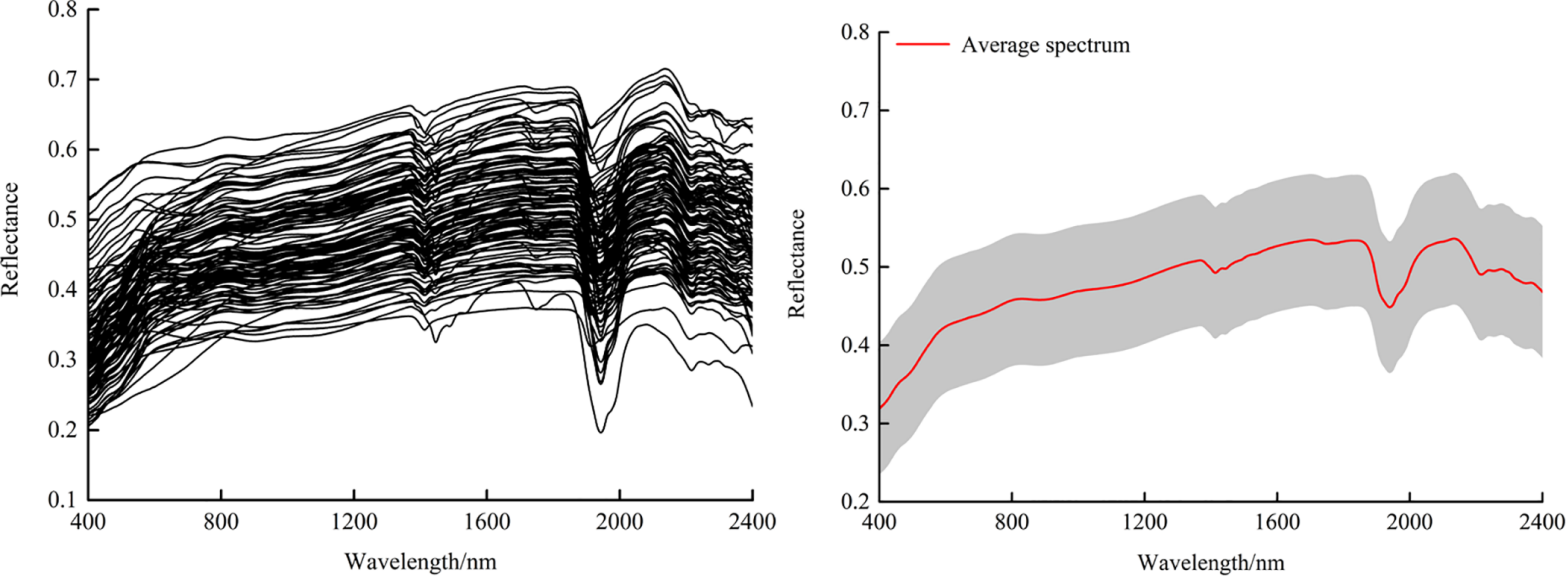
Average reflectance spectra curves and their corresponding standard deviation values (shaded regions).

The measured reflectance data were translated from binary to ASCII and exported using ViewSpecPro™ software version 6.0. Marginal wavebands with low signal-to-noise ratios (350–400 and 2401–2500 nm) were omitted in order to eliminate the noise at the edges of each spectrum [31]. Smoothing was conducted with the SG algorithm using a window size of 5 and polynomial order of 2 using OriginPro® version 9.0.0 [32]. The processed spectra constituted the final data for later analysis (S1 File). The processed spectral reflectance data of all the soil samples are illustrated in Fig 2.

Generally, absorbance spectra are employed in spectral analysis because unlike inversion (1/R), root mean square ($\sqrt{R}$), logarithm (lg R), and other forms, they have has practical spectral meaning [33]. For better modeling results and improved nonlinear relations, the previously pretreated spectral reflectance was transformed into absorbance.

### Fractional derivative

Fractional calculus is a theoretical branch of mathematics that generalizes the classic integer derivative into an arbitrary (non-integer) order. Detailed descriptions of this algorithm have been given by Schmitt [14] and by Zhang et al. [20]. In general, a fractional derivative has multiple forms, e.g., Grümwald–Letnikov (G-L), Riemann–Liouville, and Capotu [34, 35]. In order to reduce computation complexity, the G-L definition was applied to the relevant calculations [36]. The specific formula for G-L on the section is as follows:

$$d^{\alpha }f(x)=\underset{h\to 0}{\lim}\frac{1}{h^{\alpha }}\sum_{m=0}^{\left[(t-a)/h\right]}{\left(-1\right)}^{m}\frac{\Gamma (\alpha +1)}{m!\Gamma (\alpha \;\text{-}\;m+1)}f(x-mh)$$ (1)

where *α* and *h* are considered the order and step length, respectively, and the Gamma function is as defined in Eq (2):

$$\Gamma (z)=\int_{0}^{\infty }\exp(-u)u^{z-1}du=(z-1)!$$ (2)

The actual spectral resolution of the instrument in this research was 1 nm; thus, setting *h* = 1 means Eq (1) can be written as:

$$\frac{d^{\alpha }f(x)}{dx^{\alpha }}\approx f(x)+(-\alpha )f(x-1)+\frac{\left(-\alpha )(-\alpha +1\right)}{2}f(x-2)+\cdots \cdots \frac{\Gamma (-\alpha +1)}{n!\Gamma (-\alpha +n+1)}f(x-n)$$ (3)

Notably, when *α* = 1 or 2, Eq (3) is identical to the common first- and second-derivative equations. The 0.0 order stands for data that are not processed by the algorithm [20, 37, 38]. Thus, according to Eq (3), the 0.0 to the 2.0 order fractional derivatives of spectral reflectance and its absorbance (order interval: 0.2) were calculated under the Java programming integrated development platform Eclipse.

### Estimation model and prediction accuracy

#### Selection of calibration and validation set

For choosing the calibration and validation data set, the Concentration Gradient, Kennard–Stone (K-S), Sample Set Partitioning Based on Joint *x-y* Distances (SPXy), and other algorithms have been used widely [11–13]. The K-S algorithm is based on spectral distances, i.e., the spectral distance between two samples is calculated as in Eq (4). In spectral analysis, *x*_*p*_(*i*) and *x*_*q*_(*i*) are the responses at the *i*th wavelength for samples *p* and *q*:

$$d_{sp}\left(p,q\right)=\sqrt{{\sum_{i=1}^{m}\left[x_{p}\left(i\right)-x_{q}\left(i\right)\right]}^{2}}\;p,q\in \left[1,n\right]$$ (4)

The SPXy algorithm is a modification of the K-S algorithm that can accommodate multidimensional variable space and two intersample distances [39–41]. In this algorithm, the sample distances are determined based on the independent variable (*sp*) and dependent variable (*p*) space for the parameter under consideration, and *n* is the number of samples. As above, *y* means the actual clay content in this research. Therefore, the distance *d*_*p*_(*p*,*q*) can be computed as:

$$d_{p}\left(p,q\right)=\sqrt{{\left(y_{p}-y_{q}\right)}^{2}}=\left|y_{p}-y_{q}\right|\;p,q\in \left[1,n\right]$$ (5)

By assigning the same weight to the distributions of the samples in the *sp* and *p* spaces, the distances *d*_*sp*_(*p*,*q*) and *d*_*p*_(*p*,*q*) are both divided by the maximum values in the data set. Thus, the normalized *d*(*p*,*q*) can be calculated as follows:

$$d\left(p,q\right)=\frac{d_{sp}\left(p,q\right)}{\max_{p,q\in \left[1,n\right]}d_{sp}\left(p,q\right)}+\frac{d_{p}\left(p,q\right)}{\max_{p,q\in \left[1,n\right]}d_{p}\left(p,q\right)}\;p,q\in \left[1,n\right]$$ (6)

In this research, the calibration and validation data sets were selected by the SPXy algorithm, and they comprised 52 and 51 samples, respectively.

#### Modeling method and accuracy test

Partial least squares regression (PLSR) has been proven a robust and reliable approach in spectral quantitative research, primarily because of its advantages regarding dimension reduction and the synthesis and solving of collinearity problems among independent variables [20, 42]. Here, to take full advantage of spectral reflectance data, all wavelengths in the 401–2400nm range were applied in building up the models using PLSR.

The performance of clay content prediction models is often assessed by five performance indices: the ratio of performance to deviation (RPD), determinant coefficients of calibration ($R_{c}^{2}$), root mean square errors of calibration (RMSEC), determinant coefficients of prediction ($R_{p}^{2}$), and root mean square errors of prediction (RMSEP):

$$R^{2}=\frac{\sum_{i=1}^{n}\left(M_{i}-\bar{M})\cdot (P_{i}-\bar{P}\right)}{\sqrt{\sum_{i=1}^{n}{\left(M_{i}-\bar{M}\right)}^{2}\cdot \sum_{i=1}^{n}{\left(P_{i}-\bar{P}\right)}^{2}\cdot }}$$ (7)

$$RMSE=\sqrt{\frac{1}{n}\sum_{i=1}^{n}{\left(P_{i}-M_{i}\right)}^{2}}$$ (8)

RPD=SD/RMSEP (9)

where *M*_*i*_ is the measured value and *P*_*i*_ is the predicted value, $\bar{M}$ is the mean of the measured values; $\bar{P}$ is the mean of the predicted values, *SD* is the standard deviation of the measured values, and *n* is the number of samples.

Optimal models are represented by high values of $R_{c}^{2}$, $R_{p}^{2}$, and RPD but low values of RMSEC and RMSEP. Generally, the RPD can be divided into three grades: Class A (RPD ≥ 2.000) has good predictive performance; Class B (1.400 < RPD < 2.000) indicates a possibility of distinguishing between high and low levels of clay content poorly, and Class C (RPD ≤ 1.400) has no predictive ability [13, 43].

The entire calculation of this step was conducted using MATLAB^®^ software version R2012a (MathWorks, Inc., Natick, MA, USA).

## Results

### Statistical analysis of soil data

The descriptive statistical characteristics of the 103 soil samples are presented in Table 1. The clay content of all samples was low with mean and maximum values of 1.288% and 4.543%, respectively. Furthermore, the standard deviation was 0.961% and the coefficient of variation was 74.557%, indicating intermediate variability. The SOM content had a wider range, varying from 0.684 to 78.387 g kg^-1^ with a mean value of 21.429 g kg^-1^. There were significant correlations between the clay and SOM contents (*r* = 0.307), as well as clay content and EC (*r* = 0.314) at the 0.05 significance level.

**Table 1:** Statistical characteristics of various soil attributes of soil samples.

Item	Unit	Min	Max	Mean	Standard Error	Standard Deviation	Coefficient of variation	Skewness	Kurtosis
**Clay**	%	0	4.543	1.288	0.095	0.961	74.557%	1.178	1.367
**Sand**	%	1.432	70.758	23.239	1.421	14.430	62.094%	0.995	0.654
**Silt**	%	25.068	98.568	75.472	1.500	15.219	20.165%	−0.990	0.632
**SOM**	g kg^-1^	0.680	78.390	21.430	1.065	10.814	50.460%	1.336	6.148
**EC**	ms cm^-1^	0.063	84.410	18.289	2.372	23.959	131.002%	0.732	1.383
**K**^**+**^	g kg^-1^	0.031	2.922	0.378	0.036	0.367	96.953%	3.805	2.251
**Na**^**+**^	g kg^-1^	0.024	107.761	18.297	2.646	26.727	146.075%	1.590	1.517
**Ca**^**2+**^	g kg^-1^	0.104	19.900	4.326	0.459	4.634	107.125%	1.451	1.588
**Mg**^**2+**^	g kg^-1^	0.100	3.456	0.431	0.068	0.685	158.969%	2.630	7.041

### Soil spectrum

**Fig 3:**
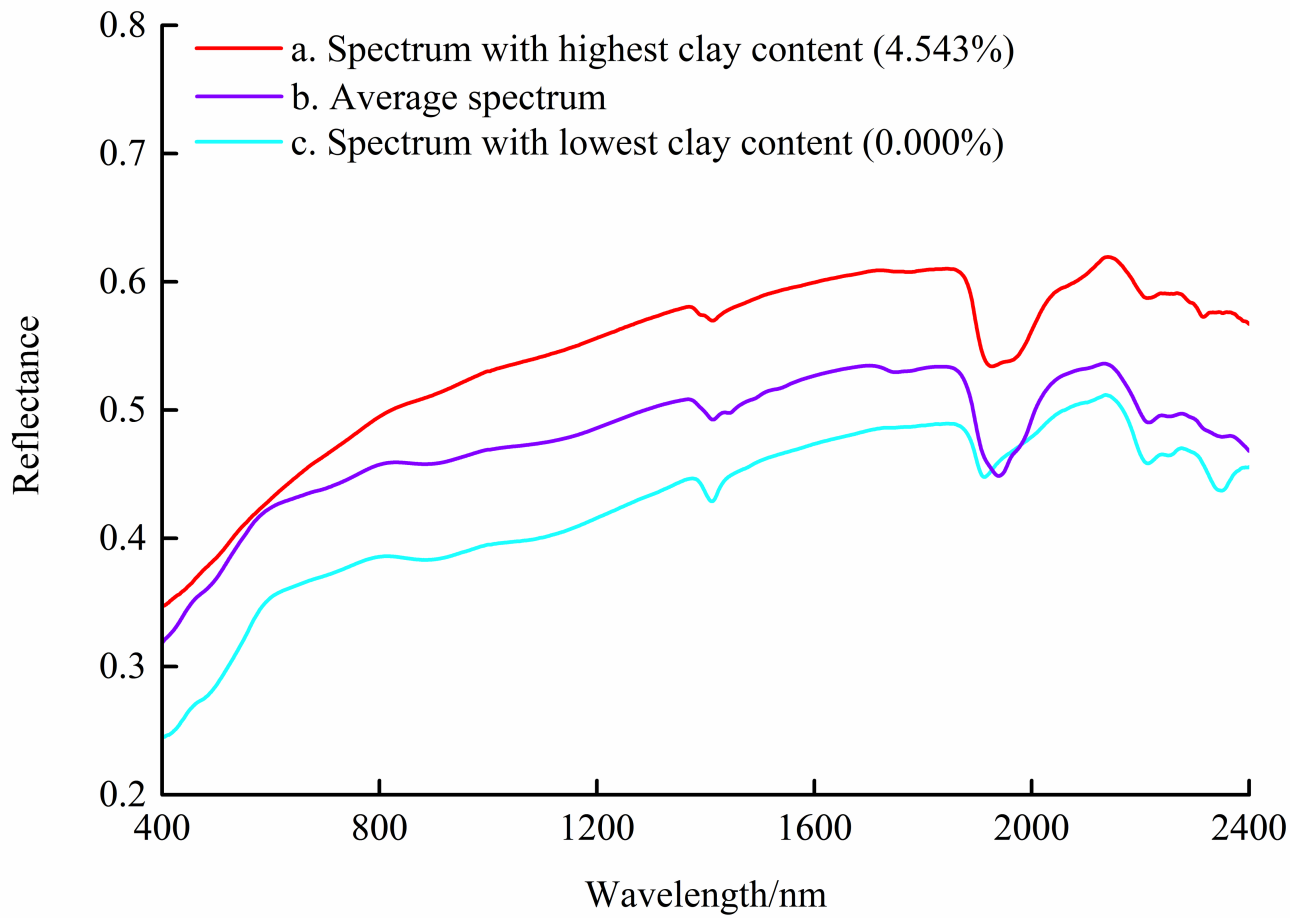
Spectral reflectance of soils with different clay contents from the Ebinur Lake basin, China. Note: spectral curve (a) denotes the soil sample with 4.543% clay content, 24.172 g kg^-1^ SOM, 68.547 g kg^-1^ Na^+^, and 6.044 g kg^-1^ Ca^2+^; spectral curve (c) denotes the soil sample with 0.000% clay content, 25.340 g kg^-1^ SOM, 3.088 g kg^-1^ Na^+^, and 2.808 g kg^-1^ Ca^2+^.

To investigate the relationship between clay content and spectral reflectance, soil samples with different clay contents were selected for curve plotting (Fig 3). The three spectral curves had similar shapes, variation tendencies and characteristic peaks. Thus, it was not complicated to distinguish the spectrum with the lowest, average, and highest clay contents in the range of 401–2400 nm, despite the spectral curves of the three soil samples having some overlapping sections (e.g., 550–600 and 1870–2000 nm). Because of moisture from different sources, there were three significant absorption features located around 1400, 1900, and 2200 nm [44, 45]. It was obvious that all three spectra had the highest reflectance near 2150 nm. The curves could be distinguished approximately from 400 to 600 nm and from 1900 to 2000 nm. The diagram shows that clay contents of 0.000% and 4.543% corresponded to the lowest and highest reflectance, respectively, with the average spectrum approximately mid-way in between. The relationship intuitively reflected the correlation of clay content and corresponding spectral reflectance, which laid the foundation for this research.

### Performance of PLSR models for quantitative estimation of clay content

Model calibration using all wavelengths can exploit all the spectral information of spectral reflectance. Furthermore, derivative pretreatment can effectively eliminate the impact of background noise on the target spectrum and enhance the spectral characteristics of the analyte [10, 46]. In order to benefit from PLSR, all raw spectral reflectance and corresponding absorbance data, pretreated by the fractional derivative, were applied in the process of model calibration. Using an order interval set to 0.2, PLSR was used to build 22 inversion models. In this research, the performances of the estimating models were affected significantly by the various derivative orders (Tables 2 and 3).

**Table 2:** Statistics of validation results of the calibration set and the corresponding performance on the validation set of raw reflectance.

Order	Principal Components	Calibration set		Validation set		
		$R_{c}^{2}$	RMSEC/%	$R_{p}^{2}$	RMSEP/%	RPD
**0.0**	2	0.417	0.927	0.254	0.869	1.033
**0.2**	2	0.306	0.925	0.423	0.863	1.090
**0.4**	2	0.323	0.905	0.517	0.832	1.149
**0.6**	2	0.539	0.862	0.465	0.788	1.130
**0.8**	3	0.538	0.848	0.530	0.770	1.179
**1.0**	3	0.459	0.872	0.551	0.772	1.196
**1.2**	4	0.671	0.713	0.741	0.639	1.482
**1.4**	4	0.809	0.643	0.706	0.576	1.400
**1.6**	4	0.723	0.700	0.729	0.615	1.458
**1.8**	5	0.907	0.425	0.916	0.364	2.484
**2.0**	5	0.905	0.445	0.880	0.388	2.103

**Table 3:** Statistics of validation results of the calibration set and the corresponding performance on the validation set of absorbance.

Order	Principal Components	Calibration set		Validation set		
		$R_{c}^{2}$	RMSEC/%	$R_{p}^{2}$	RMSEP/%	RPD
**0.0**	2	0.363	0.922	0.328	0.869	1.058
**0.2**	2	0.287	0.918	0.465	0.858	1.107
**0.4**	2	0.399	0.892	0.516	0.816	1.151
**0.6**	2	0.566	0.865	0.435	0.787	1.109
**0.8**	3	0.379	0.866	0.630	0.775	1.261
**1.0**	3	0.485	0.873	0.512	0.783	1.164
**1.2**	3	0.575	0.808	0.608	0.736	1.258
**1.4**	3	0.632	0.737	0.686	0.699	1.371
**1.6**	4	0.903	0.471	0.887	0.379	2.133
**1.8**	5	0.888	0.446	0.918	0.383	2.511
**2.0**	5	0.898	0.472	0.861	0.407	1.966

**Fig 4:**
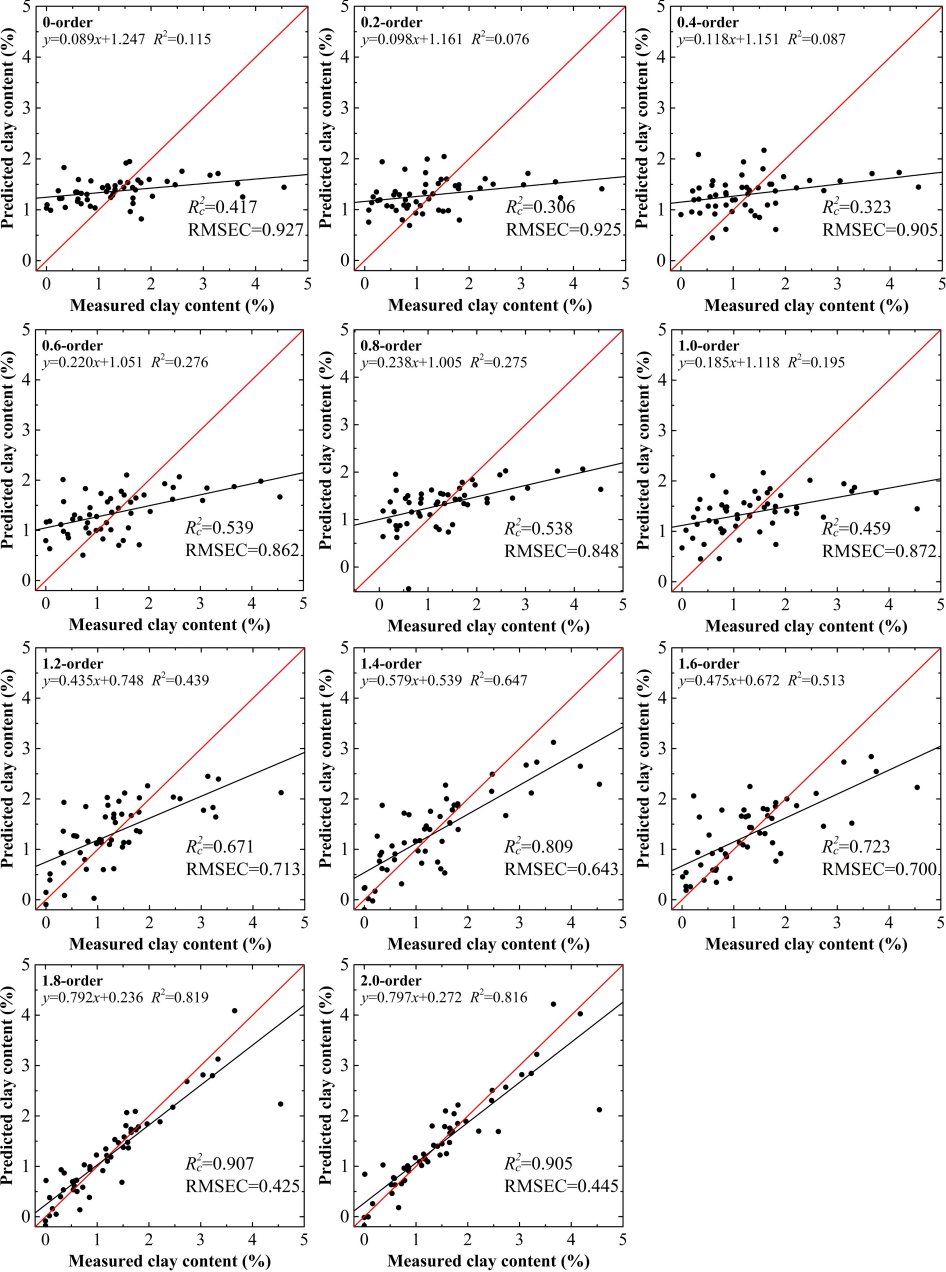
Clay content models using calibration data set based on raw spectral reflectance data treated by fractional derivatives.

**Fig 5:**
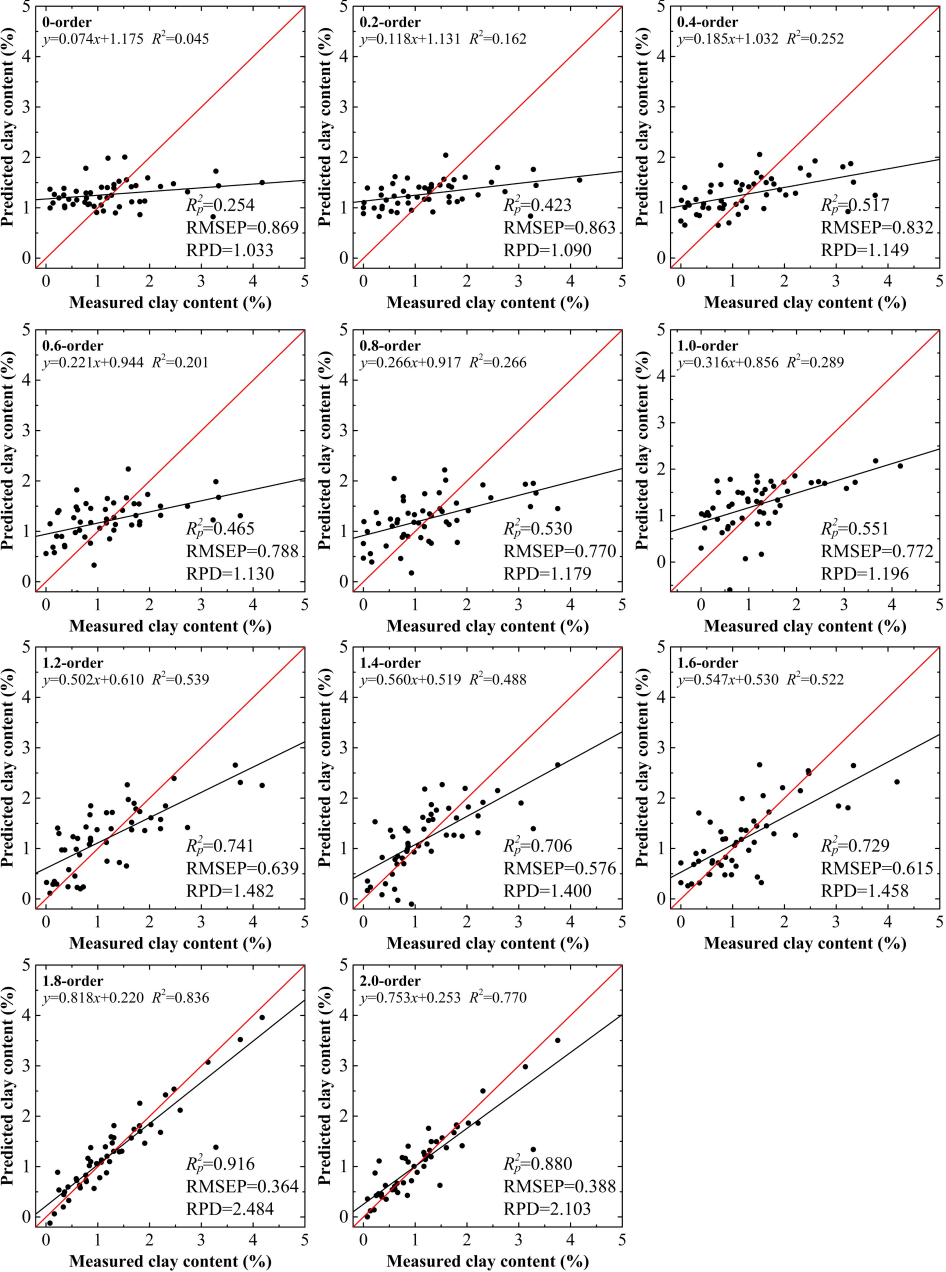
Clay content models using validation data set based on raw spectral reflectance data treated by fractional derivatives.

For spectral reflectance, in the range from the 0.0 to the 1.0 order, the trend of model preference was not obvious: the highest values of $R_{c}^{2}$, $R_{p}^{2}$, and RPD were only 0.459, 0.551, and 1.196, respectively, for the 1.0 order, while the RMSEC and RMSEP achieved their optimal status (0.848% and 0.770%, respectively) at the 0.8 order. The five parameters did not reach maximum or minimum values for the same order within the specified range. However, the indices did show a slight improvement with the increase from the 1.0 to the 1.6 order. When the order reached 1.8, the performance of the model showed significant improvement with the highest values of $R_{c}^{2}$ (0.907), $R_{p}^{2}$ (0.916), and RPD (2.484 ≥ 2.000), while the RMSEC (0.425%) and RMSEP (0.364%) were the lowest of all the 11 models. This proved to be a critical point. As the order increased to 2.0, despite higher values of RPD (≥2.000), the performance of each subsequent model was lower than the previous one (Figs 4 and 5).

**Fig 6:**
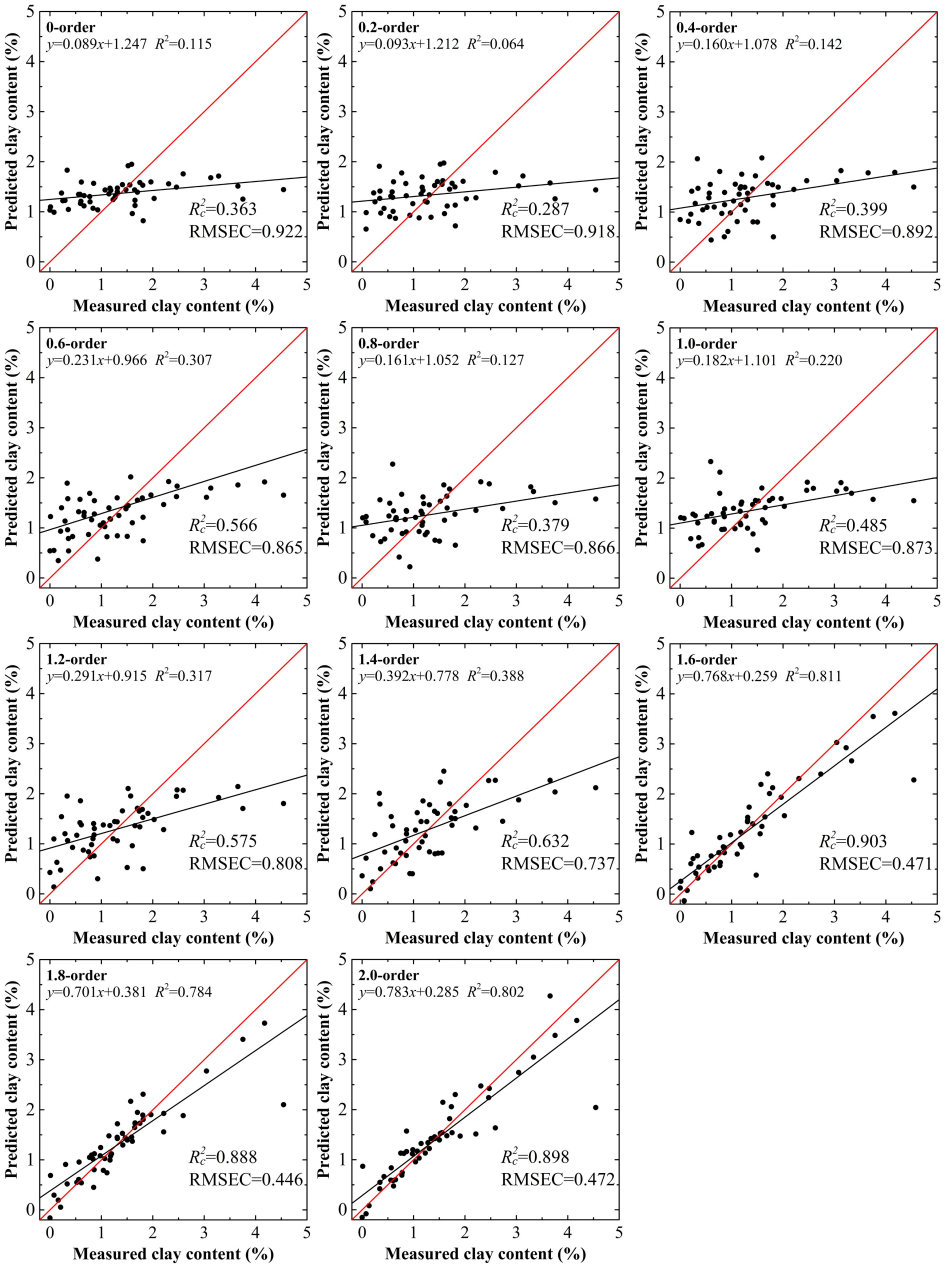
Clay content models using calibration data set based on absorbance treated by the fractional derivatives.

**Fig 7:**
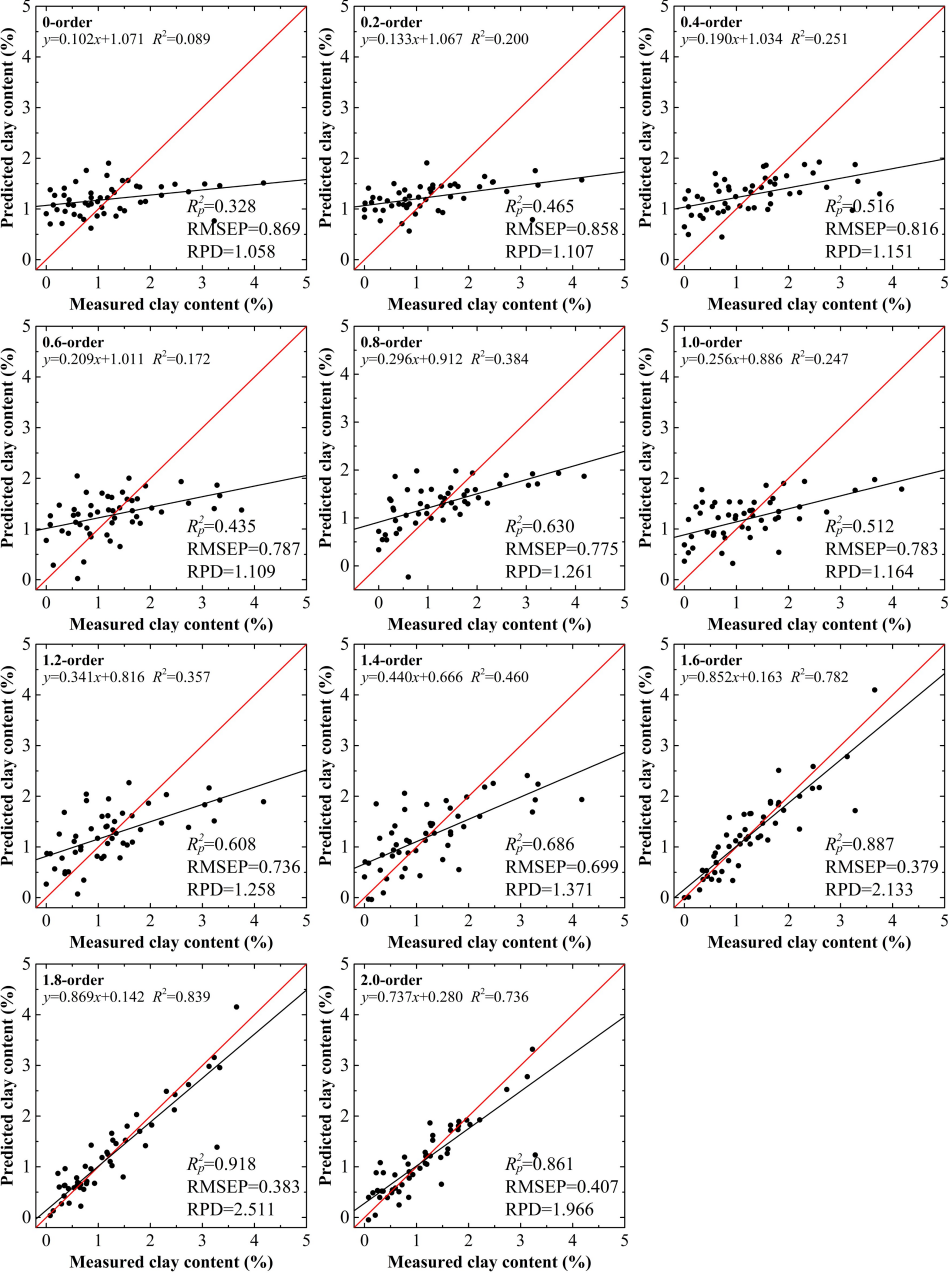
Clay content models using validation data set based on absorbance treated by the fractional derivatives.

The absorbance models built using PLSR had similar variation trends to the spectral reflectance models. The optimal accuracy parameters did not appear at the same order. Considering the case of the range from the 0.0 to the 1.2 order, the pair of $R_{c}^{2}$ and RMSEC, and the group of $R_{p}^{2}$, RMSEP, and RPD reached their optimum status at the 0.8 order and 1.2 order, respectively. For orders >1.6, the stabilities and accuracies of these models were perfected. However, despite the highest value of RPD (2.511), the model based on the 1.8 order did not possess the optimal values of $R_{c}^{2}$ and RMSEC, which were 0.903 and 0.379% at the 1.6 order, respectively. For absorbance, RPD exceeded 2.000 for two models (Figs 6 and 7). After repeated siftings to determine good predictive performance and stability, the model based on the 1.8 order was selected as the optimum inversion model of absorbance.

For clay content, the results using the validation data set with the 1.8 order were the best among the 22 models with the values of $R_{p}^{2}$ = 0.916, RMSEP = 0.364% and RPD = 2.484 and $R_{p}^{2}$ = 0.918, RMSEP = 0.383% and RPD = 2.511, for spectral reflectance and the absorbance model, respectively (Figs 5 and 7). The calibration accuracies of these two models were slightly lower than with validation data set, but they remained within a reasonable range with values of $R_{c}^{2}$ and RMSEC of 0.888–0.907, and 0.425%–0.446%, respectively. The slopes for the spectral reflectance and absorbance models with the 1.8 order using the validation data set were well distributed along the 1:1 line, indicative of good validations, while values over or under the 1:1 line indicated inaccurate estimation of the clay content in the soils of the Ebinur Lake basin (Figs 4 and 6). The results verified that the model based on spectral reflectance, pretreated using the fractional derivative, could be used to predict the clay content of soils.

## Discussion

Effective pretreatment of spectral data could enhance the features of spectral reflectance, and minimize the irrelevant and useless information of the spectra [20, 47]. Therefore, the performance of models for soil parameter estimation could be improved to some extent. The classic integer derivatives have exact physical meanings and the first and second derivatives represent the slope and curvature of the spectral curves, respectively. Normally, the order interval is 1.0, and regression models are built based on the first or second orders. However, pretreatment of the integer derivative has some disadvantages, such as spectral information loss and the introduction of high-frequency noise [14]. Compared with the integer derivative, the fractional derivative has a narrower order interval, which could reveal greater information of spectral reflectance, because the order is extended to non-integers, which could add detailed curves among the integer derivative spectral curves. Although the explicit spectral meaning of the fractional derivative has not been clarified yet, the non-local and genetic characteristics of the fractional derivate have been recognized widely. It is suggested that the fractional derivative between the 0.0 and the 2.0 order could be identified as the sensitivity to the slope and curvature of spectral curves [20]. Currently, the discrete algorithm of the fractional derivative only applies to spectral reflectance data obtained from ASD portable spectrometers with equal intervals.

Raw spectral reflectance and the first and second derivatives are three approaches commonly used in model calibration. In terms of spectral reflectance, the model using non-pretreated data (0.0 order), performed the poorest among the 11 models, with the lowest values of $R_{c}^{2}$, $R_{p}^{2}$, and RPD and the highest values of RMSEC and RMSEP (0.417, 0.254%, 1.033, 0.927%, and 0.869, respectively). For spectral reflectance pretreated by the first derivative, the performance of the corresponding model improved slightly; however, it remained inadequate for the estimation of clay content (RPD = 1.196 < 1.400). When the order was set as 2.0, the model had good prediction ability, with values of $R_{c}^{2}$ = 0.905, RMSEC = 0.445%, $R_{p}^{2}$ = 0.880, RMSEP = 0.388%, and RPD = 2.103 ≥ 2.000. When the order was extended to include non-integers, eight additional models were built based on the fractional order. Considering the five accuracy indices for these models, they did not increase or decrease directly, but rather they varied irregularly. The model based on the 1.6 order had limited predictive ability with a value of RPD = 1.458 ≥ 1.400. It is noted that the prediction ability of the model based on the 1.8 order improved with optimal values of accuracy indices (i.e., $R_{c}^{2}$ = 0.907, RMSEC = 0.425%, $R_{p}^{2}$ = 0.916, RMSEP = 0.364%, and RPD = 2.484 ≥ 2.000), which exceeded the 2.0 order model. Although the 2.0 order model has good predictive performance (RPD = 2.103 ≥ 2.000), the precision parameters of the model based on the 1.8 order had improved further to some extent. Instead of adding complexity, it was vital to obtain further modeling results and to enhance the quantitative predicting ability of the models.

Among the 11 models, 10 had better performance than the 0.0 order model and 5 performed better than the 1.0 order model. Nevertheless, only one model built on the fractional order (the 1.8 order model) was superior to the 2.0 order model. Furthermore, the variation of absorbance models showed similar trends.

In this research, the SPXy algorithm was applied to select the calibration and validation data sets. This approach is based on the distance between the independent variable and dependent variable space for the parameter under consideration [39]. Commonly, previous research has used the Concentration Gradient and K-S algorithms that consider the concentration or corresponding spectroscopy of the samples. However, the SPXy algorithm combines both these aspects and it can accommodate multidimensional variable space, e.g., the clay content and reflectance data in our study. Consequently, it was considered reasonable that these inversion models might have various calibration and validation data sets.

In previous research, clay content has been estimated quantitatively using ultraviolet–visible, VIS–NIR, and mid-infrared reflectance spectroscopy [4, 6, 48–50]. For spectral reflectance, multiple pretreating methods have been used, e.g., SG smoothing and the first derivate and second derivatives. Based on these approaches, many predicting models have been established. For example, Rossel et al. [1] applied the VIS spectral range (400–700 nm) to predict soil texture and soil organic carbon contents. Bilgili et al. [10] discovered that clay was strongly correlated with SOM, and they developed an optimized model for estimating local clay content that had good performance (*R*^2^ = 0.83, RMSE = 4.03 g kg^-1^). Using PLSR with first derivative reflectance data, Nawar et al. [11] achieved values of $R_{p}^{2}$, RMSEP, and RPD of 0.65, 8.79%, and 1.67, respectively, when predicting the clay content in the soil of El-Tina Plain in Egypt.

**Fig 8:**
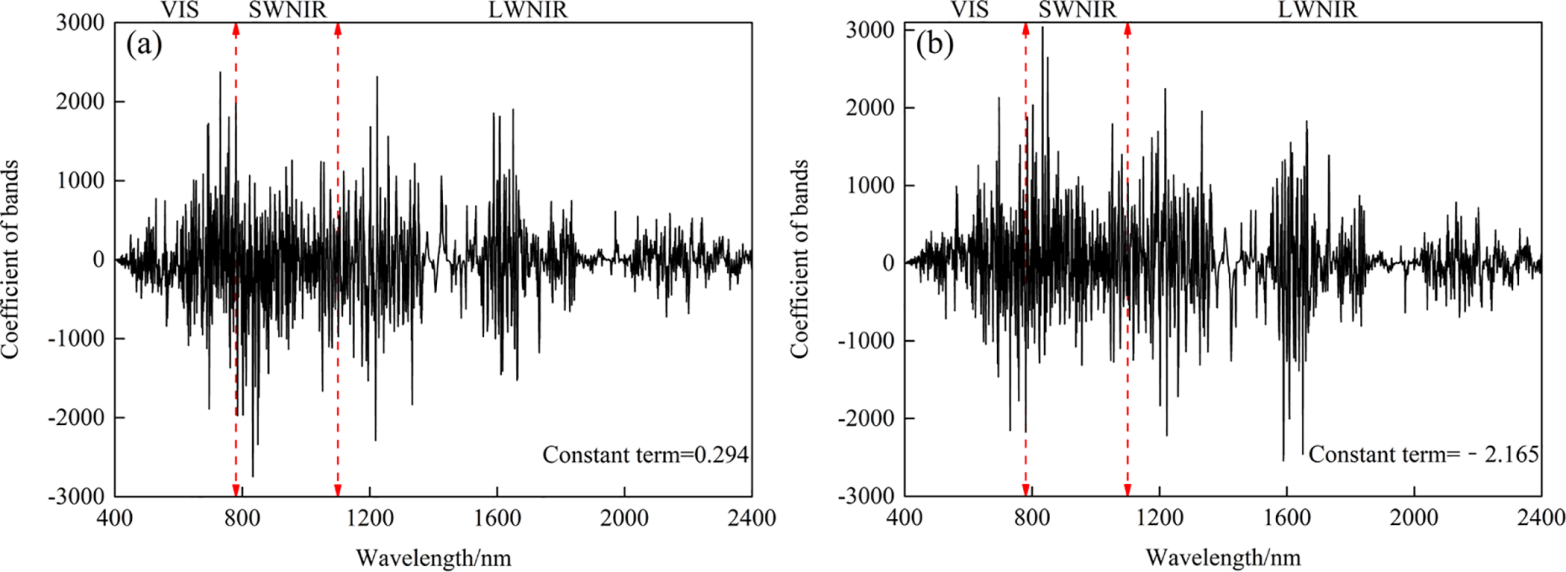
**Coefficients of all bands and the constant term of the spectral reflectance model (a) and the absorbance model (b).** Note: VIS denotes visible spectroscopy (400–780 nm), SWNIR and LWNIR denote shortwave and longwave near infrared spectroscopy (780–1100 nm and 1100–2526 nm, respectively). Red line denotes the borderline of range of VIS, SWNIR and LWNIR.

The coefficients of all wavebands and the constant term of two optimal models established in this study are illustrated in Fig 8. Results obtained in the current study were in accord with previous research, and they indicated that the relatively larger absolute values of the coefficients were located within the range 670–850 nm [51]. The use of the fractional derivative in this study allowed greater exploration of the spectral information than previous approaches; it reduced information loss, and revealed the details of the variation trends of the 5 accuracy indices based on the spectral reflectance and absorbance models of 11 orders.

In reality, only limited quantitative information can be acquired using remote sensing techniques [48]. Generally, soil spectral features are affected by variations in the SOM, EC, iron oxide, and soil texture and moisture content. The SOM content in the Ebinur Lake basin is low (near 2%). With SOM content of 2% as a boundary, that is, when SOM content exceeded 2%, the SOM played a principal role in masking out the spectral features, while the SOM content was less than 2%, it became less effective [46, 52, 53]. In the study, the soil clay content was divided into five groups: 0%–1% (*n* = 46), 1%–2% (*n* = 40), 2%–3% (*n* = 8), 3%–4% (*n* = 7), and >4% (*n* = 2). Hence, it was obvious that the clay texture was not dominant within the study area, which meant that corresponding characteristic bands were difficult to detect. In addition, the correlation between the clay content and EC was significant (*r* = 0.314). In the arid ecology, salt concentrations in soils is generally high. Soluble salts in soil could bind fine particles and further form hard salt crust, which could fix the clay of soil [54, 55]. It might influence the accuracy with clay content estimation to some degree. Furthermore, there was certain difficulty in the calibration of the retrieval model using the spectral reflectance data. The introduction of the fractional derivative algorithm generates a narrower order interval, which can reduce the loss of spectral information to some extent, extract additional spectral information, and determine the optimal prediction model. In this study, the model based on the fractional derivative 1.8 order was established as optimal.

## Conclusions

In this research, the fractional derivative algorithm was used for the pretreatment of spectral reflectance. Based on this, 22 spectral models for the estimation of clay content in the desert soils of the Ebinur Lake basin were calibrated using PLSR, and the accuracy indices of the various models were compared. It was found that the values of $R_{c}^{2}$, $R_{p}^{2}$, RMSEC, RMSEP and RPD of the models did not increase or decrease. They were irregular and they reached optimal values at a fractional order. The two best models were selected: one. calibrated based on the 1.8 order of spectral reflectance ($R_{c}^{2}$ = 0.907, RMSEC = 0.425%, $R_{p}^{2}$ = 0.916, RMSEP = 0.364%, and RPD = 2.484 ≥ 2.000), and the other based on the 1.8 order of absorbance ($R_{c}^{2}$ = 0.888, RMSEC = 0.446%, $R_{p}^{2}$ = 0.918, RMSEP = 0.383%, and RPD = 2.511 ≥ 2.000). The Ebinur Lake basin is representative of an area with severe salinization. For a model designed to predict the different clay contents of soils, the salt contents might have a certain impact on model accuracy. Therefore, the next step in future research is to distinguish the features of salt, salt ions, SOM and soil texture from spectral reflectance curves to improve estimation accuracy.

## Supporting information

S1 File The smoothed VIS–NIR spectra of 103 samples. Bands range from 401 to 2400 nm.
(XLSX)

## References

[1] Viscarra Rossel RA, Walvoort DJJ, McBratney AB, Janik LJ, Skjemstad JO. Visible, near infrared, mid infrared or combined diffuse reflectance spectroscopy for simultaneous assessment of various soil properties. Geoderma. 2006;131(1–2):59–75. doi: 10.1016/j.geoderma.2005.03.007

[2] Waiser TH, Morgan CLS, Brown DJ, Hallmark CT. In Situ Characterization of Soil Clay Content with Visible Near-Infrared Diffuse Reflectance Spectroscopy. Soil Science Society of America Journal. 2007;71(2):389 doi: 10.2136/sssaj2006.0211

[3] Jackson ML. Soil Chemical Analysis. New Delhi, India Prentice-Hall of India private limited; 1973 p. 498.

[4] Adeline KRM, Gomez C, Gorretta N, Roger JM. Predictive ability of soil properties to spectral degradation from laboratory Vis-NIR spectroscopy data. Geoderma. 2017;288:143–53. doi: 10.1016/j.geoderma.2016.11.010

[5] Gomez C, Drost APA, Roger JM. Analysis of the uncertainties affecting predictions of clay contents from VNIR/SWIR hyperspectral data. Remote Sensing of Environment. 2015;156:58–70. doi: 10.1016/j.rse.2014.09.032

[6] Shen Z, Shan Y, Peng L, Jiang Y. Mapping of Total Carbon and Clay Contents in Glacial Till Soil Using On-the-Go Near-Infrared Reflectance Spectroscopy and Partial Least Squares Regression. Pedosphere. 2013;23(3):305–11. doi: 10.1016/S1002-0160(13)60020-X

[7] Wetterlind J, Stenberg B, Jonsson A. Near infrared reflectance spectroscopy compared with soil clay and organic matter content for estimating within-field variation in N uptake in cereals. Plant and Soil. 2007;302(1–2):317–27. doi: 10.1007/s11104-007-9489-9

[8] Ben-Dor E, Banin A. Near-Infrared Analysis as a Rapid Method to Simultaneously Evaluate Several Soil Properties. Soil Science Society of America Journal. 1995;59(2):364–72. doi: 10.2136/sssaj1995.03615995005900020014x

[9] Stenberg B, Viscarra Rossel RA, Mouazen AM, Wetterlind J. Chapter Five—Visible and Near Infrared Spectroscopy in Soil Science In: Donald LS, editor. Advances in Agronomy. Volume 107: Academic Press; 2010 p. 163–215.

[10] Volkan Bilgili A, van Es HM, Akbas F, Durak A, Hively WD. Visible-near infrared reflectance spectroscopy for assessment of soil properties in a semi-arid area of Turkey. Journal of Arid Environments. 2010;74(2):229–38. doi: 10.1016/j.jaridenv.2009.08.011

[11] Nawar S, Buddenbaum H, Hill J, Kozak J, Mouazen AM. Estimating the soil clay content and organic matter by means of different calibration methods of vis-NIR diffuse reflectance spectroscopy. Soil and Tillage Research. 2016;155:510–22. doi: 10.1016/j.still.2015.07.021

[12] Shi T, Chen Y, Liu Y, Wu G. Visible and near-infrared reflectance spectroscopy—An alternative for monitoring soil contamination by heavy metals. Journal of Hazardous Materials. 2014;265:166–76. doi: 10.1016/j.jhazmat.2013.11.059 24361494 10.1016/j.jhazmat.2013.11.059

[13] Chang C, Laird DA, Mausbach MJ, Hurburgh CR. Near-Infrared Reflectance Spectroscopy–Principal Components Regression Analyses of Soil Properties Journal Paper no. J-18766 of the Iowa Agric. and Home Econ. Exp. Stn., Ames, IA. Soil Science Society of America Journal. 2001;65(2):480–90. doi: 10.2136/sssaj2001.652480x

[14] Schmitt JM. Fractional Derivative Analysis of Diffuse Reflectance Spectra. Appl Spectrosc. 1998;52(6):840–6.

[15] Arikoglu A. A new fractional derivative model for linearly viscoelastic materials and parameter identification via genetic algorithms. Rheologica Acta. 2014;53(3):219–33. doi: 10.1007/s00397-014-0758-2

[16] Machado JT, Kiryakova V, Mainardi F. Recent history of fractional calculus. Communications in Nonlinear Science and Numerical Simulation. 2011;16(3):1140–53. doi: 10.1016/j.cnsns.2010.05.027

[17] Kuldeep B, Singh VK, Kumar A, Singh GK. Design of two-channel filter bank using nature inspired optimization based fractional derivative constraints. ISA Transactions. 2015;54:101–16. doi: 10.1016/j.isatra.2014.06.005 25034647 10.1016/j.isatra.2014.06.005

[18] Tseng CC, Lee SL. Design of linear phase FIR filters using fractional derivative constraints. Signal Processing. 2012;92(5):1317–27. doi: 10.1016/j.sigpro.2011.11.030

[19] Zhang J, Chen K. Variational image registration by a total fractional-order variation model. Journal of Computational Physics. 2015;293:442–61. doi: 10.1016/j.jcp.2015.02.021

[20] Zhang D, Tiyip T, Ding J, Zhang F, Nurmemet I, Kelimu A, et al Quantitative Estimating Salt Content of Saline Soil Using Laboratory Hyperspectral Data Treated by Fractional Derivative. Journal of Spectroscopy. 2016;2016:1–11. doi: 10.1155/2016/1081674

[21] Dixon JB. Roles of clays in soils. Applied Clay Science. 1991;5(5):489–503. doi: 10.1016/0169-1317(91)90019-6

[22] He X, Lv G, Qin L, Chang S, Yang M, Yang J, et al Effects of Simulated Nitrogen Deposition on Soil Respiration in a Populus euphratica Community in the Ebinur Lake Area, a Desert Ecosystem of Northwestern China. Plos One. 2015;10(9):e0137827 doi: 10.1371/journal.pone.0137827 26379186 10.1371/journal.pone.0137827 PMC4575029

[23] Liu D, Abuduwaili J, Lei J, Wu G. Deposition Rate and Chemical Composition of the Aeolian Dust from a Bare Saline Playa, Ebinur Lake, Xinjiang, China. Water, Air, & Soil Pollution. 2011;218(1):175–84. doi: 10.1007/s11270-010-0633-4

[24] Yao J, Zhao Q, Liu Z. Effect of climate variability and human activities on runoff in the Jinghe River Basin, Northwest China. Journal of Mountain Science. 2015;12(2):358–67. doi: 10.1007/s11629-014-3087-0

[25] Abuduwailil J, ZhaoYong Z, FengQing J. Evaluation of the pollution and human health risks posed by heavy metals in the atmospheric dust in Ebinur Basin in Northwest China. Environmental science and pollution research international. 2015;22(18):14018–31. doi: 10.1007/s11356-015-4625-1 . 25956515 10.1007/s11356-015-4625-1

[26] Ge Y, Abuduwaili J, Ma L, Wu N, Liu D. Potential transport pathways of dust emanating from the playa of Ebinur Lake, Xinjiang, in arid northwest China. Atmospheric Research. 2016;178–179:196–206. doi: 10.1016/j.atmosres.2016.04.002

[27] Harmonized world soil database (version 1.1). (2009).

[28] Bao S. Soil and Agricultural Chemistry Analysis. Beijing: China Agricultural Science and Technology (In [Chinese]); 2000.

[29] Weidong L, Baret F, Xingfa G, Qingxi T, Lanfen Z, Bing Z. Relating soil surface moisture to reflectance. Remote Sensing of Environment. 2002;81(2–3):238–46. doi: 10.1016/S0034-4257(01)00347-9

[30] Liu H, Zhang Y, Zhang X, Zhang B, Song K, Wang Z, et al Quantitative Analysis of Moisture Effect on Black Soil Reflectance. Pedosphere. 2009;19(4):532–40. doi: 10.1016/S1002-0160(09)60146-6

[31] Peng X, Shi T, Song A, Chen Y, Gao W. Estimating Soil Organic Carbon Using Vis/NIR Spectroscopy with SVMR and SPA Methods. Remote Sensing. 2014;6(4):2699–717. doi: 10.3390/rs6042699

[32] Savitzky A, Golay MJE. Smoothing and Differentiation of Data by Simplified Least Squares Procedures. Analytical Chemistry. 1964;36(8):1627–39. doi: 10.1021/ac60214a047

[33] Schjønning P, McBride RA, Keller T, Obour PB. Predicting soil particle density from clay and soil organic matter contents. Geoderma. 2017;286:83–7. doi: 10.1016/j.geoderma.2016.10.020

[34] Hilfer R. Fractional Diffusion Based on Riemann-Liouville Fractional Derivatives. The Journal of Physical Chemistry B. 2000;104(16):3914–7. doi: 10.1021/jp9936289

[35] Heymans N, Podlubny I. Physical interpretation of initial conditions for fractional differential equations with Riemann-Liouville fractional derivatives. Rheologica Acta. 2006;45(5):765–71. doi: 10.1007/s00397-005-0043-5

[36] Li B, Xie W. Adaptive fractional differential approach and its application to medical image enhancement. Computers & Electrical Engineering. 2015;45:324–35. doi: 10.1016/j.compeleceng.2015.02.013

[37] Garg V, Singh K. An Improved Grunwald-Letnikov Fractional Differential Mask for Image Texture Enhancement. International Journal of Advanced Computer Sciences and Applications. 2012;3(3):130–5.

[38] Saadatmandi A, Dehghan M. A new operational matrix for solving fractional-order differential equations. Computers & Mathematics with Applications. 2010;59(3):1326–36. doi: 10.1016/j.camwa.2009.07.006

[39] Galvão RKH, Araujo MCU, José GE, Pontes MJC, Silva EC, Saldanha TCB. A method for calibration and validation subset partitioning. Talanta. 2005;67(4):736–40. doi: 10.1016/j.talanta.2005.03.025 18970233 10.1016/j.talanta.2005.03.025

[40] Insausti M, Romano C, Pistonesi MF, Band BSF. Simultaneous determination of quality parameters in biodiesel/diesel blends using synchronous fluorescence and multivariate analysis. Microchemical Journal. 2013;108:32–7. doi: 10.1016/j.microc.2012.12.007

[41] Tan C, Qin X, Li M. An ensemble method based on a self-organizing map for near-infrared spectral calibration of complex beverage samples. Analytical and Bioanalytical Chemistry. 2008;392(3):515–21. doi: 10.1007/s00216-008-2280-9 18668228 10.1007/s00216-008-2280-9

[42] Ji W, Shi Z, Huang J, Li S. In Situ Measurement of Some Soil Properties in Paddy Soil Using Visible and Near-Infrared Spectroscopy. PLoS ONE. 2014;9(8):e105708 doi: 10.1371/journal.pone.0105708 25153132 10.1371/journal.pone.0105708 PMC4143279

[43] Baldock JA, Hawke B, Sanderman J, Macdonald LM. Predicting contents of carbon and its component fractions in Australian soils from diffuse reflectance mid-infrared spectra. Soil Research. 2013;51(8):577–95. doi: 10.1071/SR13077

[44] Ackerson JP, Demattê JAM, Morgan CLS. Predicting clay content on field-moist intact tropical soils using a dried, ground VisNIR library with external parameter orthogonalization. Geoderma. 2015;259–260:196–204. doi: 10.1016/j.geoderma.2015.06.002

[45] Rossel RAV, Behrens T. Using data mining to model and interpret soil diffuse reflectance spectra. Geoderma. 2010;158(1–2):46–54. doi: 10.1016/j.geoderma.2009.12.025

[46] Jin X, Du J, Liu H, Wang Z, Song K. Remote estimation of soil organic matter content in the Sanjiang Plain, Northest China: The optimal band algorithm versus the GRA-ANN model. Agricultural and Forest Meteorology. 2016;218–219:250–60. doi: 10.1016/j.agrformet.2015.12.062

[47] Rinnan Å, Berg Fvd, Engelsen SB. Review of the most common pre-processing techniques for near-infrared spectra. TrAC Trends in Analytical Chemistry. 2009;28(10):1201–22. doi: 10.1016/j.trac.2009.07.007

[48] Ramasamy V, Anandalakshmi K. The determination of kaolinite clay content in limestones of western Tamil Nadu by methylene blue adsorption using UV–vis spectroscopy. Spectrochimica Acta Part A: Molecular and Biomolecular Spectroscopy. 2008;70(1):25–9. doi: 10.1016/j.saa.2007.07.008 17884718 10.1016/j.saa.2007.07.008

[49] Peng Y, Knadel M, Gislum R, Schelde K, Thomsen A, Greve MH. Quantification of SOC and Clay Content Using Visible Near-Infrared Reflectance–Mid-Infrared Reflectance Spectroscopy With Jack-Knifing Partial Least Squares Regression. Soil Science. 2014;179(7):325–32. doi: 10.1097/ss.0000000000000074

[50] Islam K, Singh B, McBratney A. Simultaneous estimation of several soil properties by ultra-violet, visible, and near-infrared reflectance spectroscopy. Soil Research. 2003;41(6):1101–14. doi: https://doi.org/10.1071/SR02137

[51] Sankey JB, Brown DJ, Bernard ML, Lawrence RL. Comparing local vs. global visible and near-infrared (VisNIR) diffuse reflectance spectroscopy (DRS) calibrations for the prediction of soil clay, organic C and inorganic C. Geoderma. 2008;148(2):149–58. doi: 10.1016/j.geoderma.2008.09.019

[52] Wang J, Tiyip T, Ding J, Zhang D, Liu W, Wang F. Quantitative Estimation of Organic Matter Content in Arid Soil Using Vis-NIR Spectroscopy Preprocessed by Fractional Derivative. Journal of Spectroscopy. 2017;2017:1–9. doi: 10.1155/2017/1375158 10.1371/journal.pone.0184836 PMC5608292 28934274

[53] Shi Z, Wang Q, Peng J, Ji W, Liu H, Li X, et al Development of a national VNIR soil-spectral library for soil classification and prediction of organic matter concentrations. Science China Earth Sciences. 2014;57(7):1671–80. doi: 10.1007/s11430-013-4808-x

[54] Fujimaki H, Shimano T, Inoue M, Nakane K. Effect of a Salt Crust on Evaporation from a Bare Saline Soil. Vadose Zone Journal. 2006;5(4):1246–56. doi: 10.2136/vzj2005.0144

[55] Han W, Ma Z, Lai Z, Appel E, Fang X, Yu L. Wind erosion on the north-eastern Tibetan Plateau: constraints from OSL and U-Th dating of playa salt crust in the Qaidam Basin. Earth Surface Processes and Landforms. 2014;39(6):779–89. doi: 10.1002/esp.3483

